# Identification of De Novo JAK2 and MAPK7 Mutations Related to Autism Spectrum Disorder Using Whole-Exome Sequencing in a Chinese Child and Adolescent Trio-Based Sample

**DOI:** 10.1007/s12031-019-01456-z

**Published:** 2019-12-14

**Authors:** Jian Jiao, Manxue Zhang, Pingyuan Yang, Yan Huang, Xiao Hu, Jia Cai, Chan Yang, Mingjing Situ, Hui Zhang, Lei Fu, Kuifang Guo, Yi Huang

**Affiliations:** 1 Mental Health Center, West China Hospital of Sichuan University, Chengdu, China; 2 Psychiatric Laboratory, State Key Laboratory of Biotherapy, West China Hospital, Sichuan University, Chengdu, Sichuan China; 3 Brain Research Center, West China Hospital of Sichuan University, Chengdu, China

**Keywords:** Autism spectrum disorder, De novo single-nucleotide variations, *JAK2*, *MAPK7*, Whole-exome sequencing

## Abstract

Autism spectrum disorder (ASD) is a neurodevelopmental disorder with high phenotypic and genetic heterogeneity. Whole-exome sequencing studies have shown that de novo single-nucleotide variations (SNVs) play an important role in sporadic ASD. The present study aimed to search for de novo SNVs using whole-exome sequencing in 59 unrelated Chinese ASD sporadic trios, and found 24 genes (including five reported ASD candidate genes *CACNA1D*, *ACHE*, *YY1*, *TTN*, and *FBXO11*) with de novo harmful SNVs. Five genes (*CACNA1D*, *JAK2*, *ACHE*, *MAPK7*, and *PRKAG2*) classified as “medium-confidence” genes were found to be related to ASD using the Phenolyzer gene analysis tool, which predicts the correlation between the candidate genes and the ASD phenotype. De novo SNVs in *JAK2*, *MAPK7*, and *PRKAG2* were first found in ASD. Both *JAK2* and *MAPK7* were involved in the regulation of the MAPK signaling pathway. Gene co-expression and inter-gene interaction networks were constructed and gene expression data in different brain regions were further extracted, revealing that *JAK2* and *MAPK7* genes were associated with certain previously reported ASD genes and played an important role in early brain development. The findings of this study suggest that the aforementioned five reported ASD genes and *JAK2* and *MAPK7* may be related to ASD susceptibility. Further investigations of expression studies in cellular and animal models are needed to explore the mechanism underlying the involvement of *JAK2* and *MAPK7* in ASD.

**Electronic supplementary material:**

The online version of this article (10.1007/s12031-019-01456-z) contains supplementary material, which is available to authorized users.

## Introduction

Autism spectrum disorder (ASD) is a lifelong neurodevelopmental condition with a prevalence of 1–2% of the general population (Lai et al. 2014; Lord et al. 2018). According to the *Diagnostic and Statistical Manual of Mental Disorders, Fifth Edition* (DSM-V), the ASD category includes autistic disorder, Asperger’s disorder, pervasive developmental disorders not otherwise specified, and childhood disintegrative disorder (Lord et al. 2018). The core symptoms of this disease are persistent deficits in social communication and restricted, repetitive sensory-motor behaviors (Lord et al. 2018). A substantial portion of individuals with ASD require lifelong support, thus constituting a huge burden for both families and society (Lavelle et al. 2014).

Various pieces of evidence have indicated that genetics plays an important role in the etiological mechanism of ASD. Consistency rates in monozygotic twins, dizygotic twins, and siblings of 30–99%, 0–65%, and 3–30%, respectively, have been found, with an estimated overall heritability of 0.7–0.8 (Bailey et al. 1995; Rosenberg et al. 2009; Hallmayer et al. 2011; Colvert et al. 2015). Early genetic studies focused on identifying multiple genetic lineages to support genetic linkage analysis aimed at identifying chromosomal regions commonly inherited by patients with the disease. However, only two loci (one in chromosome 20p13 and the other in chromosome 7q35) reached statistical significance across the genome and could be replicated in different studies (Alarcon et al. 2002; Weiss et al. 2009; Werling et al. 2014). Based on this, quantitative phenotypes (language delay and social responsiveness) were found to be related to *CNTNAP2* on chromosome 7q35 (Alarcon et al. 2002; Chiocchetti et al. 2015). However, this method was less efficient in complex diseases due to the combined effects of many genetic and environmental factors (Sener et al. 2016). The genome-wide association study (GWAS), as a powerful data-driven approach for identifying common variants with low penetrance, permits an unbiased and comprehensive scan for susceptibility genes and has greater statistical power than the linkage study (Liu et al. 2014). This method was used to assess the contribution of common variants to ASD, revealing that they accounted for 40–60% of the total deficits (Gaugler et al. 2014). As this method requires a large sample size, only *CDH9* and *CDH10* on 5p14.1 and *MACROD2* on 20p12.1 reached genome-wide significance (Wang et al. 2009; Anney et al. 2010; Ramaswami et al. 2018). However, these results have not been replicated in other studies. Many small-scale chromosomal abnormalities have been found by array comparative genomic hybridization and microarray techniques, confirming that the copy number variation (CNV) is closely related to ASD. Based on this, de novo CNVs, such as duplication of 7q11.23 and microdeletion of 16p11.2, were found to be recurrent in patients with ASD (Sanders et al. 2011; Blumenthal et al. 2014). Nevertheless, many genes with CNVs located at noncoding regions and the sizes of de novo CNVs exhibited large differences, which involved excessive heterogeneity, making it difficult to determine the role of a specific gene in the disease.

Relative to the noncoding region, the exon region represents less than 1% of the human genome but contains 85% of known disease-causing genetic variants (Sener et al. 2016). High-throughput sequencing technology has made it possible to detect de novo or rare point mutations in the coding region in genes. Whole-exome sequencing (WES) technology is used to identify the variations in all coding regions of genes, and to some extent it overcomes the research deficiencies mentioned earlier (Sener et al. 2016). Another advantage is that specific genes with genetic variations can be discovered in sporadic cases, and the relevant biological pathways can be further studied (Sener et al. 2016). Since 2011, WES has led to the identification of many new susceptibility genes with de novo single-nucleotide variations (SNVs), which play an important role in sporadic cases of ASD. It has been estimated that 400–1000 genes may be related to ASD (Geschwind et al. 2015). Despite its high heritability, ASD is genetically complex, and the underlying genetic architecture is still not well understood (Schaaf et al. 2011; Liu et al. 2014). Notably, one study found that only 13% of de novo missense mutations in the coding region of genes contributed to 12% of diagnoses (Iossifov et al. 2014), implying that more than 80% of de novo missense mutations were not true ASD pathogenic mutations and may not be related to the ASD phenotype. Therefore, the selection of de novo missense mutations has become a problem requiring further investigation. Relevant software programs (e.g., SIFT [Sorting Intolerant From Tolerant], MutationTaster) have been used to assess the harmfulness and conservation of gene mutation sites (Thongnak et al. 2018). Other software tools, such as RVIS [Residual Variation Intolerance Score] (Ronemus et al. 2014) and TADA [Transmission And De novo Association] (Li et al. 2016), which prioritize the list of genes depending on the impact of mutations and assess the tolerance of genes to genetic mutations, have also been used. However, the relationship between genes and phenotypes has not been analyzed or predicted. Moreover, although several studies have conducted WES in ASD on relatively large sample sizes and performed functional verification at the cellular level based on the de novo SNVs found in the special genes (Sadybekov et al. 2017; Wen et al. 2017), few studies have reported on a multiple-expression level, such as multigene co-expression, inter-gene interaction, or gene expression analysis in the brain region, to verify the role of de novo SNVs in the phenotypes of ASD. Therefore, in the present study, WES was conducted in 59 family trios with a child with ASD; de novo SNVs were selected using comprehensive bioinformatics and multiple-expression analysis to confirm the results.

## Methods

### Patient Recruitment and Demographic Characteristics

The patients were recruited from the second outpatient department of the West China Mental Health Center of Sichuan University, special education schools, and nursery and primary schools in Chengdu city, in addition to online recruitment. A child psychiatrist used the DSM-V criteria to make a preliminary clinical diagnosis in the children, and the patients with ASD were then diagnosed independently by two well-trained child psychiatrists using the Autism Diagnostic Interview-Revised (ADIR) (Lord et al. 1994) and Autism Diagnostic Observational Schedule (ADOS) (Lord et al. 1989). Any patient who had definite somatic or hereditary disease, including congenital heart disease, Rett syndrome, Down syndrome, fragile X syndrome, phenylketonuria, or epilepsy, was excluded. Parents who had no other relatives diagnosed with ASD within three family generations signed informed consent for genetic testing. Eventually, a total of 59 ASD trios participated in this study (probands: male/female = 57:2, ADIR: 40.05 ± 16.26, ADOS: 18.31 ± 4.79, intelligence quotient: 84.11 ± 23.65, age: 8.71 ± 3.05). The study protocol was approved by the Ethics Committee of the West China Hospital and was conducted in accordance with the ethical guidelines of the Declaration of Helsinki.

### Whole-Exome Sequencing

Blood was obtained from all members of the 59 trios using an ethylenediaminetetraacetic acid (EDTA) anticoagulation tube, and genomic DNA was extracted from the whole blood. DNA degradation and suspected RNA/protein contamination were verified by electrophoresis in 1% agarose gel. The DNA sample concentration and purity were further precisely quantified using the Qubit dsDNA HS Assay Kit in the Qubit 3.0 Fluorometer (Life Technologies/Thermo Fisher Scientific, Waltham, MA, USA). The exome sequences were efficiently enriched from 0.4-μg genomic DNA, which was required for library generation using the Agilent SureSelect Human All Exon V6 liquid capture system (Agilent Technologies, Santa Clara, CA USA), according to the manufacturer’s protocol. The DNA library was sequenced on an Illumina HiSeq 4000 system (Illumina, Inc., San Diego, CA, USA) for paired-end 150 bp reads.

### Data Processing and Variant Calling

Quality control was followed by filtering reads with adapter contamination (>10 nucleotides not aligned to the adapter, allowing ≤10% mismatches) and discarding a paired read if >10% of bases were uncertain in either read so as to guarantee a meaningful downstream analysis. Paired reads in which any of the single reads had more than 50% low-quality (Phred quality <5) nucleotides were also discarded. Valid sequencing data were mapped to the reference genome (GRCh37/hg19) using the Burrows-Wheeler Aligner (BWA) software to obtain the original mapping result in the BAM format. Subsequently, SAMtool and Picard (http://broadinstitute.github.io/picard/), which are software programs using internationally accepted filtering criteria, were respectively used to sort bam files and perform duplicate marking to generate the final bam file. These duplicate reads were uninformative and not considered as evidence for variants. The Picard was employed to mark these duplicates so that they could be ignored in the following analysis. After this process, SAMtools, mpileup, and bcftools were used for variant calling and identification of SNVs.

### Functional Annotation and Filter

To further examine the association between genetic variation and disease, ANNOVAR [ANNOtate VARiation] (Wang et al. 2010) was used to functionally annotate variations that were obtained in the previous steps. The variant position, variant type, conservative prediction, and other information were obtained from several databases, including dbSNP [Single Nucleotide Polymorphism Database] (http://www.ncbi.nlm.nih.gov/projects/SNP/), the International Genome Sample Resource from the 1000 Genomes Project (http://www.1000genomes.org/), ExAC [Exome Aggregation Consortium] (http://exac.broadinstitute.org/), HGMD [Human Gene Mutation Database] (http://www.hgmd.cf.ac.uk/ac/index.php/), and CADD [Combined Annotation-Dependent Depletion] (https://cadd.gs.washington.edu/). As this study was focused on the exonic variants, gene transcript annotation databases, including Consensus Coding Sequence Project (CDS), RefSeq [NCBI Reference Sequence Database], Ensembl, and the University of California, Santa Cruz (UCSC) Genome Browser, were also applied for annotation to determine the amino acid alteration. Variants obtained from previous steps were then filtered with the minor allele frequency > 1% in the 1000 Genomes databases (1000 Genomes Project Consortium). Only rare SNVs occurring in exons or in canonical splice sites (splicing junction 10 bp) were further analyzed to investigate the amino acid changes. Harmful mutations in all nonsynonymous SNVs were retained using the SIFT (Ng et al. 2003), MutationTaster (Schwarz et al. 2010), and gerp++gt2 (Davydov et al. 2010) software to categorize the detrimental mutation types.

### Primers and Sanger Sequencing Validation

As WES is performed by fragment sequencing, the sequencing error rate is relatively high (Ku et al. 2012), and it is important to further validate the identified de novo variants using Sanger sequencing. As nonsynonymous and harmful variants of genes are likely to be associated with true ASD candidate genes (Hnoonual et al. 2016; Miryounesi et al. 2019), the gene harmful mutation sites were verified using Sanger sequencing. These mutation sites were as follows: *ACHE* (c.G1165A: p. E389K); *TMUB2* (c.G112A: p. V38I); *YY1* (c.G961A: p.G321S); *AOC1* (c.C1687T: p. R563C); *PRAG1* (c.C2783T: p. S928F); *KIF15* (c.T796A: p. L266I; c.G832A: p. E278K); *JAK2* (c.G649C: p. V217L); *MAPK* (c.C709T: p.R237C); *RNF31* (c.C1624T: p. R542C); *CACNA1D* (c.C4127T: p. T1376M); *C1GALT1* (c.T620C: p. M207T); *QSER1* (c.C2788G: p. L930V); *CELSR3* (c.C1865T: p. A622V); *ZNF276* (c.G584T: p. C195F); *VPS9D1* (c.G742C: p. D248H); *ADGRL3* (c.T4075C: p. F1359L); *WDR63* (c.C764T: p. T255M); *PRKAG2* (c.C221T: p. P74L); *MCMDC2* (c.T1532C: p. L511P); *AASDH* (c.G638T: p. R213L); *TTN* (c.G26999A: p. R9000H); *FBXO11* (c.G2335A: p. A779T); *HIST1H2AG* (c.G245C: p. R82P); ZNF512 (c.C1069T: p. P357); *HIPK1* (c.G81C: p. E27D); and *EXTL3* (c.G1504A: p. D502N). Polymerase chain reaction (PCR) assays and Sanger sequencing were performed. The primers used in this study are listed in Table S1.

### Selection of SNVs/Genes

The criteria for SNV/gene selection included the combination of the strength of the association between gene and phenotype and the involvement of genes in neural synaptic formation and brain development. Genes containing de novo harmful mutations with the strongest association with ASD phenotype were screened out using the Phenolyzer (Yang et al. 2015) (http://phenolyzer.wglab.org/), which integrates information from disease, gene–disease, and gene–gene relational databases to analyze the correlation between the mutant gene and the disease phenotype. When entering a disease name (such as “autism spectrum disorder”) in the “Diseases/Phenotypes,” the Phenolyzer generates a confidence score based on the correspondence of each gene to the disease name by determining all the genes that have a reported association with the disease (seed genes) in query-precompiled databases (Yang et al. 2015). Then, “gene selection” is clicked, and the candidate genes, which carry the nonsynonymous and harmful variants (see above in “Primers” and “Sanger sequencing validation”) obtained from WES sequencing in the pop-up box, are entered. The seed genes are then expanded to the candidate genes based on several types of gene–gene relationship logic to include related genes, such as displaying protein–protein interactions, gene families, transcriptional regulation, or regulation by another gene (Yang et al. 2015). Finally, all the information is integrated to generate a score for the candidate genes, with the weights trained from a logistic regression model (http://phenolyzer.wglab.org/FAQ.php). The negative intercept, meaning the genes not associated with the ASD phenotype, was deducted to ensure that the final gene score was positive. The scores were renormalized to the final prioritized gene list. The software workflow can be found at http://phenolyzer.wglab.org/FAQ.php. The association between genes and phenotypes were defined based on the Phenolyzer score for the gene: low confidence (<0.1), medium confidence (0.1 ≤ Phenolyzer score < 0.5), and high confidence (≥0.5) (Fang et al. 2017). The higher the score, the stronger the association between genes and phenotypes. Genes strongly associated with the phenotypes are the focus of further research.

### Pathway Enrichment and the Expression of Genes in the Brain

To exclude genes not expressed in the brain, the PubMed Gene database (https://www.ncbi.nlm.nih.gov/gene/), which contains information about the expression of genes in different tissues and disease information related to the gene, was used. The genes expressed in the brain with a gene-phenotype score > 0.1 as defined by the Phenolyzer are the targeted genes, as these genes are the most closely associated with the ASD phenotype. As the function of ASD-related genes is focused mostly on neurodevelopment and synapse formation (De Rubeis et al. 2014; Hammerschlag et al. 2019), the STRING database (https://string-db.org/), a system for predicting protein interactions to extract pathway information and functional information, was used to further explore whether the genes with a gene-phenotype score > 0.1 were involved in the neural synaptic formation and developmental pathway. In addition, expression data for genes associated with neural-synaptic formation and developmental pathways in different brain regions and developmental stages were extracted using the HBT [Human Brain Transcriptome] database, which contains transcriptome data and associated metadata for the developing and adult human brain.

### Building the Network of Gene Co-Expression and Genetic Interaction

**Fig. 1:**
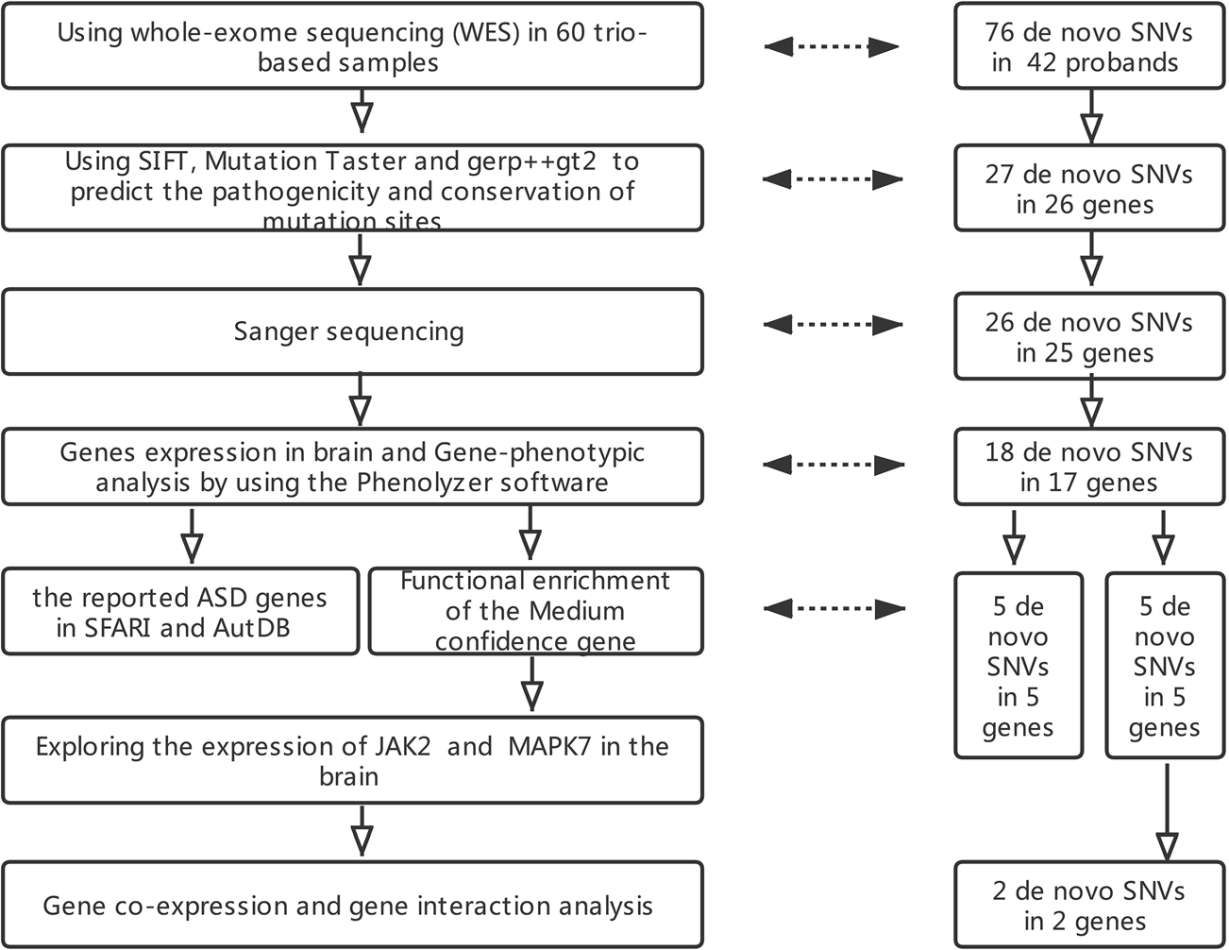
Overall experimental procedure and gene screening steps

The functional association and co-expression relationship between candidate genes found in this study and previously reported ASD genes were investigated by performing gene co-expression and gene interaction analysis. Again, the network of gene co-expression was built using spatially and temporally rich transcriptome data extracted from the BrainSpan database (http://www.brainspan.org/), which is a database of gene expression in specific brain regions obtained from RNA sequencing and exon microarray techniques. Pearson correlation coefficients (*r*) were then used to represent the index of the gene co-expression level. To avoid missing genes that were reported, all the genes in the “Human Gene Module” in SFARI Gene (https://gene.sfari.org/) and in “Human Gene” in AutDB (http://autism.mindspec.org/) (Basu et al. 2009) were used. Both are centers of susceptibility genes implicated in ASD, providing an up-to-date and annotated list of ASD candidate genes in the form of a reference data set for interrogating molecular mechanisms underlying the disorder (Basu et al. 2009). The reported ASD genes exhibited strong co-expression correlation coefficients (absolute *r* ≥ 0.6) with candidate genes extracted in this study. The data set used to explore the association between candidate genes and previously reported ASD genes was obtained from GeneMANIA (http://genemania.org/) (Warde-Farley et al. 2010) using the Cytoscape program plugin (Smoot et al. 2011), which is a web interface that can display an interactive functional association network between genes and data sets, to create a genetic interaction network. In addition, a number of nodes (representing genes) and edges (representing the interaction of two genes) between the candidate genes and previously reported ASD genes were calculated using GeneMANIA. Both networks were drawn and visualized by employing the Cytoscape program (overall experimental procedure and gene screening steps in Fig. 1).

## Results

### Detection of De Novo Mutations

Each sample had approximately 9.71–22.42 Gb of cleaned sequencing data obtained after removing the adapters and low-quality bases (Data S1). Overall, each person had more than 99.77% of reads aligned to the reference genome (GRCh37/hg19), and after the removal of PCR duplications, at least 54% effective reads were obtained from the target regions. More than 99.7% of target regions had at least tenfold coverage, and more than 99.4% and 79.6% of the target regions had 20- and 50-fold coverage, respectively.

A total of 76 de novo SNVs (56 missense mutations, 10 unknown mutations, 5 non-frameshift deletions, 3 frameshift deletions, 1 stop-gain mutation, and 1 synonymous mutation) were found in 42 probands (Data S2). Using the software (SIFT, MutationTaster, and gerp++gt2) to analyze the harmfulness and conservation of the mutation site, it was found that only 27 de novo SNVs were possible pathogenic sites left in the 26 genes. PCR and Sanger sequencing were further conducted for validation and found that the mutation site in the *WDR63* gene was false positive and, therefore, was excluded. Finally, 26 de novo harmful SNVs in 25 gene coding regions of 19 probands were retained (listed in Data S3).

### Analysis and Identification of the De Novo Gene Mutations in ASD

**Fig. 2:**
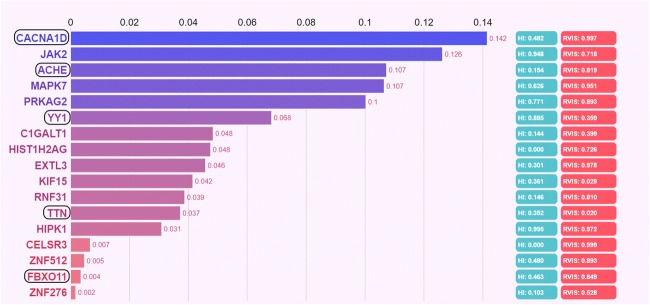
Prioritizing candidate genes using the Phenolyzer. The genes tagged with the boxes were ASD-related genes found in the SFARI Gene database and the AutDB. The first column is the gene score, ranging from 0 to 1; the greater the score, the stronger the association with the disease. HI represents the haploinsufficiency score. The score range is 0–1; the larger the score, the greater the possibility of haploinsufficiency. RVIS represents the Residual Variation Intolerance Score. This is used to describe gene tolerance, ranging from 0 to 1; the greater the score, the worse the tolerance

**Fig. 3:**
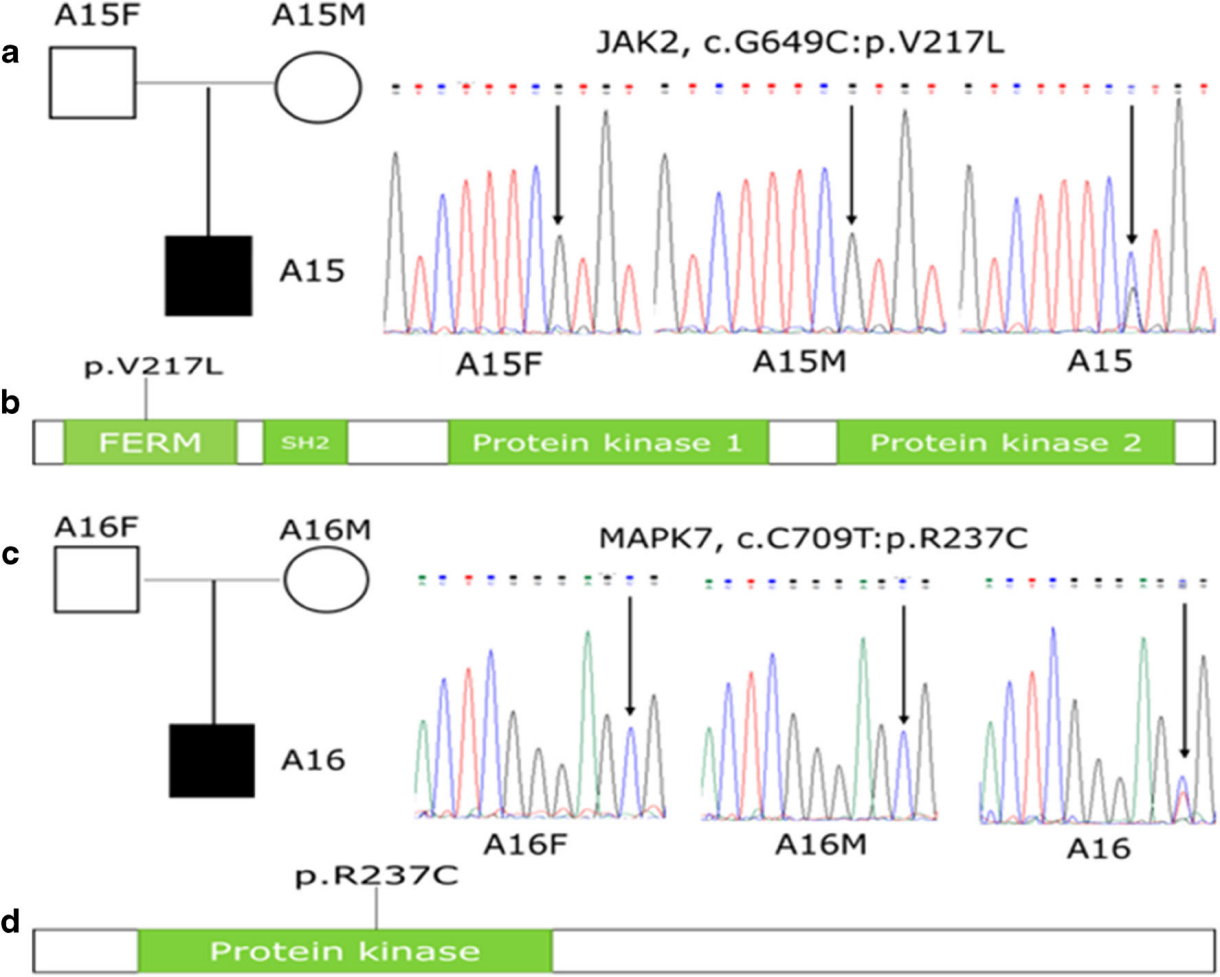
De novo mutations (DNM) and relative positions in *JAK2* and *MAPK7*. **a** The DNM of *JAK2* (c. G649C: p. V217L) was confirmed by Sanger sequencing in A15. **b** Schematic representation of the *JAK2* protein. **c** The DNM of *MAPK7* (c.C709T: p. R237C) was confirmed by Sanger sequencing in A16. **d** Schematic representation of the *MAPK7* protein. Black represents amino acid changes caused by mutations in these two gene loci in patients with ASD

Twenty-four of the 25 genes were found to be expressed in the brain, the exception being the *AOC1* gene. Of these 24, only 17 genes were shown by Phenolyzer analysis to be associated with the ASD phenotypes (Fig. 2). The other seven genes (*TMUB2*, *PRAG1*, *QSER1*, *VPS9D1*, *ADGRL3*, *MCMDC2*, and *AASDH*) were excluded because of the negative intercept, which means that these genes may not be associated with the ASD phenotype. Analysis using the SFARI and AutDB database further showed that these seven genes were also not previously reported in ASD. A search of PubMed Gene (https://www.ncbi.nlm.nih.gov/gene/) additionally showed that none of these seven genes were reported in any other mental disorders. However, five (*CACNA1D*, *ACHE*, *YY1*, *TTN*, and *FBXO11*) of the remaining 17 genes listed in Fig. 2 were found in the SFARI and AutDB databases, and their mutation sites had never been reported (Table 1). Five genes (*CACNA1D*, *JAK2*, *ACHE*, *MAPK7*, and *PRKAG2*) were classified as medium-confidence genes, which meant that these genes were strongly associated with the disease phenotypes (Fig. 2). Using the STRING database for function enrichment, the molecular function of all five gene products was found to be ion-binding. The *ACHE*, *CACNA1D*, and *JAK2* genes were also found to take part in the formation of a cholinergic synapse, while the *CACNA1D* and *MAPK7* genes were involved in the regulation of the MAPK signaling pathway (Data S4). The *JAK2* gene was further found through PubMed (https://www.ncbi.nlm.nih.gov/gene/3717) to participate in the MAPK cascade process, which played an important role in the MAPK signaling pathway. All five genes were clearly predicted to be functionally deleterious by these prediction tools (Table 1). However, de novo SNVs in *JAK2* and *MAPK7* were first discovered in ASD, and both these genes are involved in the regulation of the MAPK signaling pathway. *JAK2* (c. G649C: p. V217 L), located in the FERM domain of the gene (Fig. 3a and b), was found in proband A15. *MAPK7* (c.C709T: p. R237C), located in the protein kinase domain of the gene (Fig. 3c and d), was found in proband A16.

**Table 1:** Summary of seven de novo SNVs detected by trio-based WES of ASD, with five of those genes having been reported in ASD before

Family	Chrom ^†^	Gene	Func ^‡^	ExonicFunc^§^	Mutation	AAChang	SIFT	MutationTaster	gerp++gt2	Reported (genes/sites)
A 1	7	*ACHE*	Exonic	Missense SNV	c.G1165A	p. E389K	Damaging	Damaging	Conserved	Yes/No
A18	3	*CACNA1D*	Exonic	Missense SNV	c.C4127T	p. T1376 M	Damaging	Damaging	Conserved	Yes/No
A49	2	*TTN*	Exonic	Missense SNV	c.G26999A	p. R9000H	Damaging	Damaging	Conserved	Yes/No
A51	2	*FBXO11*	Exonic	Missense SNV	c.G2335A	p. A779T	Damaging	Damaging	Conserved	Yes/No
A4	14	*YY1*	Exonic	Missense SNV	c.G961A	p. G321S	Damaging	Damaging	Conserved	Yes/No
A15	9	*JAK2*	Exonic	Missense SNV	c.G649C	p. V217 L	Damaging	Damaging	Conserved	No/No
A16	17	*MAPK7*	Exonic	Missense SNV	c.C709T	p. R237C	Damaging	Damaging	Conserved	No/No

Using hg19 as the human reference genome

Chrom ^†^: Chromosome. Func ^‡^: Comment on the area where the mutation site is located; the exonic should include the coding exonic portion, UTR3, and UTR5, but the ANNOVAR comment indicates that the exonic represents only the coding exonic portion. ExonicFunc ^§^: SNV variant type of exon region

### Expression Profile of *JAK2* and *MAPK7* in the Human Brain

Using the HBT database, expression data were extracted for genes in different brain regions and developmental stages and indicated that *JAK2* and *MAPK7* were widely expressed in all human brain regions in different developmental stages. It is worth noting that both *JAK2* and *MAKP7* demonstrated distinct peak-level expression during the middle and late stages of pregnancy (Fig. S1). More importantly, the *JAK2* gene reached its highest level of expression in the cerebellar cortex (CBC) and remained relatively stable in the later trajectory of life. The expression of *MAPK7* gradually reduced after birth, while the changes in the expression level were nearly the same in different brain regions (Fig. S1).

### Co-Expression and Genetic Interaction Network Analyses of *JAK2* and *MAPK7*

**Fig. 4:**
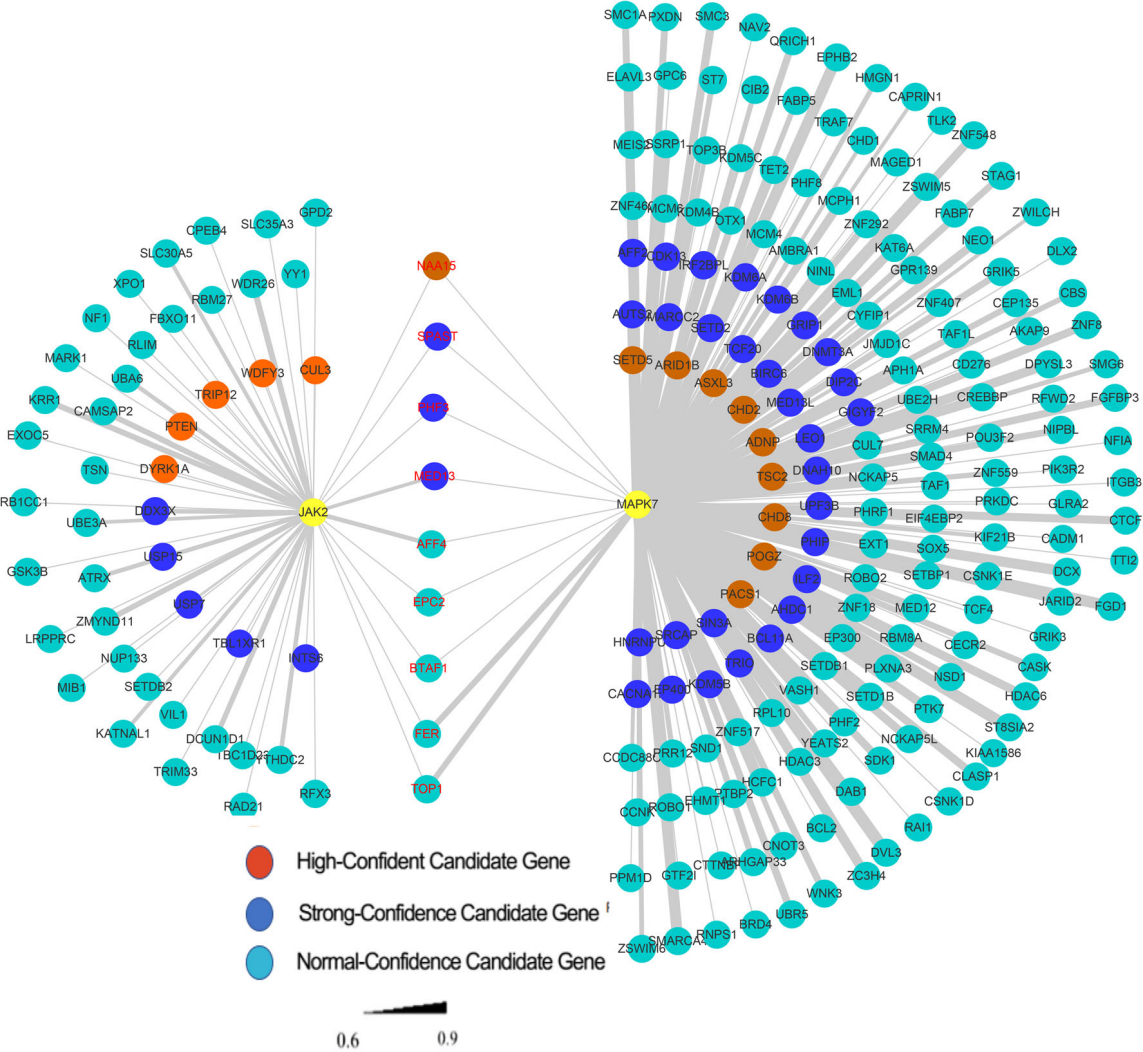
Gene co-expression network analysis of *JAK2* and *MAPK7*. Pearson correlation coefficients (*r*) were used to represent the gene co-expression levels between each pair of genes. The orange-red circles represent high-confidence candidate genes in the SFARI Gene database and genes (evidence score: 5 stars) in the AutDb database. The blue circles represent strong-confidence candidate genes in the SFARI database and genes (evidence score: 4 stars) in the AutDb database. The green circles represent the remaining genes in the two gene databases

The relationship between the *JAK2* and *MAPK7* genes and the previously reported ASD candidate genes was explored using the BrainSpan database to extract gene co-expression data. *JAK2* was co-expressed with 53 previously reported ASD candidate genes, including six high-confidence genes (achieving genome-wide statistical significance with independent replication) (Fig. 4) and eight strong-confidence genes (achieving genome-wide statistical significance with independent replication, but with standards slightly relaxed from those for high-confidence genes); the gene scoring process can be found at https://gene.sfari.org/about-gene-scoring/criteria/. *MAPK7* was co-expressed with 183 previously reported ASD candidate genes, including 10 high-confidence genes and 32 strong-confidence genes (Fig. 4). Moreover, nine previously reported ASD candidate genes, including one high-confidence gene (*NAA15*) and three strong-confidence genes (*MED13, SPAST,* and *PHF3*), simultaneously appeared in the co-expression network of *JAK2* and *MAPK7*, indicating the presence of functional interactions between them.

Using the GeneMANIA database to extract data on inter-gene interaction between *JAK2*, *MAPK7*, and the previously reported ASD candidate genes, *JAK2* was found to have an interaction relationship with five previously reported ASD candidate genes (*DDX1*, *BRD4*, *GNB1L*, *BRCA2*, and *OXTR*), while *MAPK7* was observed to interact with three previously reported ASD candidate genes (*DLX1*, *DMXL2*, and *CX3CR1*) (Fig. S2). All eight of these previously reported ASD candidate genes were nominated as normal-confidence candidate genes in the SFARI Gene database, indicating that they were validated in independent replication but did not achieve genome-wide statistical significance.

## Discussion

In this study, WES was performed among 59 sporadic trios, and 24 genes with de novo harmful SNVs were found to be expressed in the brain. The Phenolyzer (Yang et al. 2015), a software tool that reveals hidden associations between genotypes and phenotypes, was used to validate the correlation between the originally known genes with de novo SNVs and the disease phenotype by combining prior biological knowledge and phenotype information from different databases (Disease Ontology, Online Mendelian In Man, GWAS Catalog and Human Protein Relation Database, etc.). Five genes (*CACNA1D*, *JAK2*, *ACHE*, *MAPK7*, and *PRKAG2*) were found to be “medium-confidence” genes related to ASD. De novo SNVs in *JAK2*, *MAPK7*, and *PRKAG2* were first found in ASD. Both *JAK2* and *MAPK7* were involved in the regulation of the MAPK signaling pathway, which took part in the neural-synaptic formation and brain development. This study involved multiple verification steps, including gene co-expression, inter-gene interaction analysis, and early gene expression profile in the brain, to further validate the association between *JAK2* and *MAPK7* mutations and ASD susceptibility. This study was novel in illustrating the important role of *JAK2* and *MAPK7* in the etiology of ASD in the same sample at the gene–gene interaction level and gene expression levels.

Through gene co-expression and inter-gene interaction network analysis, many previously reported ASD genes were found to be in the *JAK2* and *MAPK7* co-expression and interaction networks, several of which co-existed in the co-expression network of both genes. Among the genes co-existing in the co-expression network, one (*NAA15*) and three genes (*MED13*, *SPAST*, *PHF3*) were listed as “high-confidence” and “strong-confidence” genes, respectively, in the SFARI Gene database. Multiple different mutation sites in these genes have been reported by various studies in patients with ASD, suggesting that *JAK2* and *MAPK7* may act as a functional link between these co-expressing genes. However, due to significant genetic heterogeneity, only several previously reported ASD candidate genes (*DDX1*, *BRD4*, *GNB1L*, *BRCA2*, *OXTR* and *DLX1*, *DMXL2*, and *CX3CR1*) were found to exist in the *JAK2* and *MAPK7* gene interaction networks. These results indicate that *JAK2* and *MAPK7* may interact with the aforementioned genes at the functional level of the protein. Therefore, the interaction between *JAK2*, *MAPK7*, and the known ASD-related genes provide further evidence that ASD is caused by these genetic variants.

Previous studies have shown that enlarged head circumference is a ubiquitous phenomenon in children with ASD, which is due to an increased rate of brain growth before the age of 2 years (Hazlett et al. 2011). Moreover, dynamic macrostructural and microstructural changes from the mid-fetal stage to 2 years after birth are also closely related to ASD (Ouyang et al. 2019), indicating that neurodevelopmental conditions and dysplasia in the fetal and infant stages play a crucial role in the pathogenesis of ASD. Using HBT software, this study found that both *JAK2* and *MAPK7* genes were expressed in multiple developmental stages in different brain regions and reached the peak of their expression in the mid–late embryonic stage, which is the crucial developmental period of the brain with ASD. Furthermore, the *JAK2* gene was found to have the highest expression level in the CBC, playing a central role in cognitive and emotional processing, which are key deficits in autism and other neuropsychiatric disorders (Menashe et al. 2013; Wang et al. 2014). The degrees of variation in expression levels in different brain regions are similar for *MAPK7*, and the peak expression levels of this gene appear in the mid-embryo phase, which involves a rapid increase in the volume of the cortical plate and the surface of the human brain (Clouchoux et al. 2012; Andescavage et al. 2017). The changes in cortical thickness, especially the volume gain of the gray matter during brain development associated with language development, social cognition, and behavioral control, are driven by a lack of typical age-related increase in cortical thickness and play an important role in early childhood autism (Smith et al. 2016). The findings of this study indicate that *JAK2* and *MAPK7* play a role in early brain development, and their mutations may result in minor structural deficits, eventually causing ASD-related symptoms.

Chromosome fragment abnormalities are closely related to ASD, including 7q abnormalities (Alarcon et al. 2002) and 9p24.1 microdeletion (Kantojärvi et al. 2010). In proband A16, the only nonsynonymous and harmful mutation was *MAPK7* (c.C709T: p. R237C), located in the protein kinase domain of the gene (Fig. 3c and d). *MAPK7* is located in chromosome 17p11.2, where a 3.7-Mb duplication exists, which is considered the cause of Potocki-Lupski syndrome (PTLS; MIM #610883), a disease having approximately 70–90% comorbidity with ASD (Lacaria et al. 2012). *RAI1* is one of the important pathogenic genes in this region (Abad et al. 2018). Rare de novo mutations in the *RAI1* gene have been found to be closely related to ASD (Abad et al. 2018), and animal models showed that the haploinsufficiency of this gene was associated with social abnormalities in mice (Rao et al. 2017). As illustrated in this study, *MAPK7* had a co-expression relationship with *RAI1*. It is speculated that *MAPK7* may participate in the etiology of ASD. This gene also takes part in the MAPK signaling pathway. In the nervous system, the brain-derived neurotrophic factor stimulates neural differentiation and survival of human umbilical cord blood mesenchymal stem cells via the MAPK signaling pathway (Lim et al. 2008). Many genes in this pathway are involved in the pathogenesis of many psychiatric disorders, such as ASD (Wen et al. 2016), attention deficit and hyperactivity disorder (ADHD), bipolar disorder, and schizophrenia (Zhao et al. 2018). Moreover, the protein encoded by *MAPK7* is a member of the MAPK family involved in a wide variety of cellular processes, such as proliferation, differentiation, transcription regulation, and brain development (Pearson et al. 2001), and abnormalities in these processes can lead to the development of ASD symptoms (Nagy et al. 2017; Courchesne et al. 2019). Previous evidence also indicated rare mutations in the *MAPK* gene family participating in ASD, such as *MAPK3* (Park et al. 2017) and *MAPK1* knockout mice showing ASD-like behavior (Satoh et al. 2011). Together with these results of de novo SNVs in *MAPK7*, it may be inferred from this study that mutations in the MAPK family may involve the genes that affect multiple cellular processes, resulting in the inability of cells to develop and mature normally, leading to the onset of ASD.

*JAK2* is located in chromosome 9p24.1 where deletions have been found in patients with ASD in earlier studies (Kantojärvi et al. 2010). The nonsynonymous and harmful de novo SNV of *JAK2* (c. G649C: p. V217L) found in this study was located in the FERM domain of the gene (Fig. 3a and b), which encodes the cytoskeletal-associated proteins that constitute a link between the membrane and the cytoskeleton involved in the signal transduction pathways. Evidence from functional studies suggest that *JAK2* is involved in the JAK-STAT cascade, a process that includes the development, maintenance, and survival of central nervous system glial cells and neurons as well as enhanced brain-derived neurotrophic factor expression (Kaur et al. 2005; Kazim et al. 2015). Previous studies have demonstrated that JAK-STAT activation signals play a crucial role in ASD immune dysfunction (Ahmad et al. 2017), and it was found to participate in the MAPK cascade process (https://www.ncbi.nlm.nih.gov/gene/3717) in the MAPK signaling pathway. Furthermore, it was found that abnormal synaptic functions, especially in the basal forebrain cholinergic dysfunction, may lead to ASD (Garber et al. 2007). Along with the findings using the STRING database, the present study also found that *JAK2* participated in the cholinergic synapse. Therefore, *JAK2* may be involved in the genetic etiological mechanism of ASD.

This study had several limitations. First, no association analysis was performed in an independent case–control sample on these two de novo SNVs. However, multiple verification methods, including Phenolyzer software, which combines many different databases to integrate all known information in order to more accurately analyze the correlation between the mutant gene and the disease phenotype, were used to overcome the limitations of the sample size. Second, the study did not validate the newly discovered ASD candidate genes at the expression level. However, with the use of the STRING, BrainSpan, GeneMANIA, and HBT databases to extract the co-expressed and inter-gene interaction data, as well as gene expression profile data in the brain, the link between *JAK2*, *MAPK7*, and ASD was verified at multiple levels of gene interaction and expression using the most advanced databases. Future studies on gene expression in cells and animal models are still needed to explore the precise function of *JAK2* and *MAPK7* in ASD.

This study found the de novo SNVs of *JAK2* and *MAPK7* genes in the MAPK signaling pathway to be related to ASD from different perspectives, including relationships between genes and phenotypes, gene co-expression, gene interaction network analysis, and gene expression in early brain development. The results suggest that *JAK2* and *MAPK7* genes in the MAPK signaling pathway may play an important role in the etiology of ASD through interaction with other ASD-related genes and eventually lead to the abnormal development of the ASD brain. This information may shed new light on the genetic etiology and therapeutic drugs in ASD. Further genetic and functional studies are needed to elucidate the precise molecular mechanisms involved in the joint action of multiple genes.

## Electronic supplementary material

ESM 1 (DOCX 317 kb)

ESM 2 (XLSX 99 kb)

ESM 3 (XLSX 11 kb)
