# Supplementary material for: Identification of De Novo JAK2 and MAPK7 Mutations Related to Autism Spectrum Disorder Using Whole-Exome Sequencing in a Chinese Child and Adolescent Trio-Based Sample
Source: J Mol Neurosci. 2019 Dec 14;70(2):219–29. doi: 10.1007/s12031-019-01456-z (PMC7018782; doi:10.1007/s12031-019-01456-z)
Supplement: Supplementary file 1 — (DOCX 317 kb) [file 12031_2019_1456_MOESM1_ESM.docx]

FIGURE S1

b

a


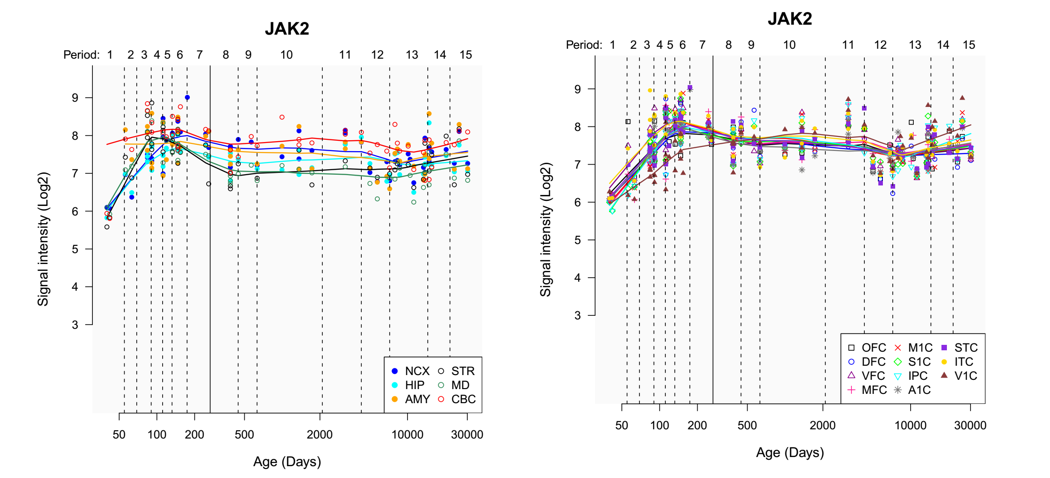


c d


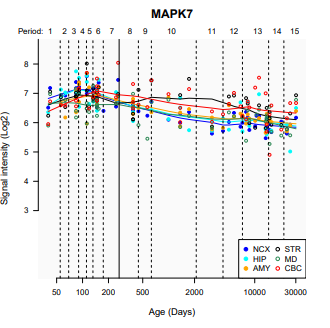

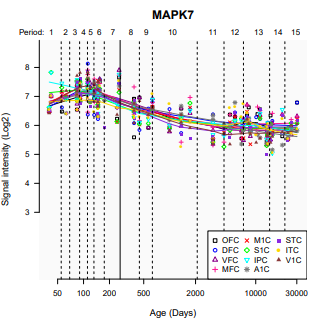


FIGURE S1. Expression analysis of JAK2 and MAPK7 in 6 human brain regions (a, c) (including cerebellar cortex (CBC), mediodorsal nucleus of the thalamus (MD), striatum (STR), amygdala (AMY), hippocampus (HIP) and 11 areas of neocortex (b, d). In both figures, there is a solid line represents the normal delivery time. OFC: orbital prefrontal cortex, DFC: dorsolateral prefrontal cortex, VFC: ventrolateral prefrontal cortex, MFC: medial prefrontal cortex, M1C: primary motor cortex, S1C: primary somatosensory cortex, IPC: posterior inferior parietal cortex, A1C: primary auditory cortex, STC: posterior superior temporal cortex, ITC: inferior temporal cortex, V1C: primary visual cortex

FIGURE S2


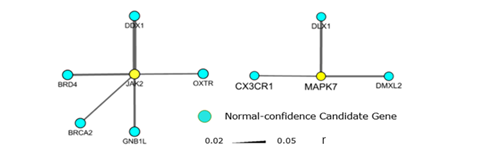


FIGURE S2. Gene-interaction network analysis analysis of *JAK2* and *MAPK7*. The Person correlation coefficients ( r ) were reprent the gene-interaction levels between each pair of genes. Candidate ASD genes were extracted from the SFARI database and the AutDb database. Green circle represent the normal-confidence genes in the SFARI database and genes (Evidence score: 1-3 stars) in the AutDb database.
